# COGNITIVE ASPECTS OF MANAGING STRESS AND UNWANTED EMOTION SUCCESSFULLY IN OLD AGE

**DOI:** 10.1093/geroni/igad104.0109

**Published:** 2023-12-21

**Authors:** Tammy English

## Abstract

The ability to cope with stressors and manage unwanted emotion is central to health-related outcomes across adulthood. Prominent models of adult development suggest there may be age-related shifts in motivational strivings and accumulated knowledge that help support emotional well-being in old age. At the same time, older adults have cognitive and physical vulnerabilities that may offset some of these strengths. This session will showcase new work leveraging experimental and experiencing sampling methods to examine how people of varying ages and resource levels manage their emotions. These studies provide new insights into how older adults respond to stressors and adverse stimuli in controlled settings and daily life contexts. The first talk centers on how individuals are negatively impacted by cognitive stressors regardless of age, but older adults show more resilience than younger adults after stressor exposure. The second talk focuses on how a cognitive process known as affective labeling (or naming one’s emotions) can create emotion regulation difficulties, especially in old age. The third talk examines emotion regulation success and the benefits of instructional support for cognitively normal older adults compared to those with mild cognitive impairment (MCI). The fourth talk extends this work by tracking emotion regulation motives in daily life, showing that cognitively diverse older adults report fewer attempts to manage their emotions, but similar reasons for doing so, compared to relatively younger adults. Together these presentations delineate age-related similarity and differences in emotion regulation, highlighting avenues through which coping capacity may be maintained and improve in late life.

